# Expression and Function of the Lipocalin-2 (24p3/NGAL) Receptor in Rodent and Human Intestinal Epithelia

**DOI:** 10.1371/journal.pone.0071586

**Published:** 2013-08-05

**Authors:** Christian Langelueddecke, Eleni Roussa, Robert A. Fenton, Frank Thévenod

**Affiliations:** 1 Institute of Physiology & Pathophysiology, ZBAF, University of Witten, Herdecke, Witten, Germany; 2 Anatomy and Cell Biology II, Albert-Ludwigs-University Freiburg, Freiburg, Germany; 3 Department of Biomedicine and InterPrET Center, Aarhus University, Aarhus C, Denmark; National Institute of Agronomic Research, France

## Abstract

The lipocalin 2//NGAL/24p3 receptor (NGAL-R/24p3-R) is expressed in rodent distal nephron where it mediates protein endocytosis. The mechanisms of apical endocytosis and transcytosis of proteins and peptides in the intestine are poorly understood. In the present study, the expression and localization of rodent 24p3-R (r24p3-R) and human NGAL-R (hNGAL-R) was investigated in intestinal segments by immunofluorescence and confocal laser scanning microscopy, immunohistochemistry and immunoblotting. r24p3-R/hNGAL-R was also studied in human Caco-2 BBE cells and CHO cells transiently transfected with r24p3-R by immunofluorescence microscopy, RT-PCR and immunoblotting of plasma membrane enriched vesicles (PM). To assay function, endocytosis/transcytosis of putative ligands phytochelatin (PC_3_), metallothionein (MT) and transferrin (Tf) was assayed by measuring internalization of fluorescence-labelled ligands in Caco-2 BBE cells grown on plastic or as monolayers on Transwell inserts. The binding affinity of Alexa 488-PC_3_ to colon-like Caco-2 BBE PM was quantified by microscale thermophoresis (MST). r24p3-R/hNGAL-R expression was detected apically in all intestinal segments but showed the highest expression in ileum and colon. Colon-like, but not duodenum-like, Caco-2 BBE cells expressed hNGAL-R on their surface. Colon-like Caco-2 BBE cells or r24p3-R transfected CHO cells internalized fluorescence-labelled PC_3_ or MT with half-maximal saturation at submicromolar concentrations. Uptake of PC_3_ and MT (0.7 µM) by Caco-2 BBE cells was partially blocked by hNGAL (500 pM) and an *EC*_*50*_ of 18.6 ± 12.2 nM was determined for binding of Alexa 488-PC_3_ to PM vesicles by MST. Transwell experiments showed rapid (0.5-2 h) apical uptake and basolateral delivery of fluorescent PC_3_/MT/Tf (0.7 µM). Apical uptake of ligands was significantly blocked by 500 pM hNGAL. hNGAL-R dependent uptake was more prominent with MT but transcytosis efficiency was reduced compared to PC_3_ and Tf. Hence, r24p3-R/hNGAL-R may represent a high-affinity multi-ligand receptor for apical internalization and transcytosis of intact proteins/peptides by the lower intestine.

## Introduction

Little is known about the transepithelial transport and absorption of proteins in the intestine. Neonates have the ability to absorb immunoglobulins from the intestine as a means of passive immunization [1,2]. Furthermore, viruses, such as HIV, may infect the host by transcytosis across the intestinal mucosa [3]. To a very limited extent, the adult mammalian small intestine is capable of transcytosis of a variety of food substances and environmental contaminants to a very limited extent [4]. Moreover, non-digested dietary components, such as plant components, can be degraded in the ileum and large intestine by microbial fermentation and serve as a source of energy and nutrients for host metabolism [5,6]. Once the complex carbohydrates of the plant wall have been broken down by the intestinal microbiota, released plant proteins may be reabsorbed or undergo proteolysis by the large intestine microbiota [7]. For example, a significant part of plant-derived toxic cadmium-bound phytochelatins (PCs) and metallothioneins (MTs) are absorbed intact by enterocytes and are found subsequently in the kidney [8,9].

In contrast to the lack of *in vivo* data on mucosal protein transcytosis, *in vitro* cell models have been established to study protein transcytosis, e.g. in the human Caco-2 cell line, and the processes of apical endocytosis, trafficking and transcytosis have been well characterized [10–12]. Interestingly, a receptor for apical-to-basolateral intestinal transport of proteins and peptides has not be specifically looked for [1], though the multi-ligand receptor complex megalin/cubilin/amnionless has been detected in the brush border of mammalian terminal ileum [13,14]. In this intestinal segment it is thought to mediate apical internalization of specific proteins, such as the intrinsic factor-vitamin B12 complex [15] or transferrin (Tf) [16]. In addition, apical Tf uptake and transcytosis has been linked to a Tf receptor *in vitro* [11,17,18].

In the kidney, apical receptor-mediated-endocytosis (RME) of proteins and peptides has been well characterized. Plasma proteins, which are filtered by the glomeruli, are almost completely reabsorbed by the tubular system [19]. The majority of filtered proteins are reabsorbed in the proximal tubule (PT) by RME via the multiligand high-capacity receptor complex megalin/cubilin/amnionless [20,21] and degraded in lysosomes. The contribution of the distal nephron to protein reabsorption varies depending on various factors and ranges between 3–25% of filtered proteins [22,23]. We have recently implicated a novel receptor, the lipocalin-2 (24p3/neutrophil gelatinase-associated lipocalin (NGAL)) receptor (r24p3-R), in protein endocytosis by the distal nephron [24]. The r24p3-R/r24p3 ligand complex has been previously associated with the regulation of iron uptake and apoptosis [25]. Further putative roles of the 24p3-R/r24p3 ligand complex include an antibacterial innate immune response [26,27] and epithelial tissue regeneration following kidney ischemia-reperfusion injury [28]. The r24p3-R is expressed in the apical membranes of rodent distal tubules and collecting ducts [24]. Furthermore, experiments in cultured cells indicated that proteins, such as albumin, transferrin and MT are high-affinity ligands of the r24p3-R, which mediates RME of these proteins [24].

The aim of this study was to elucidate r24p3-R/hNGAL-R expression and localization in intestinal segments and to investigate the role of hNGAL-R in the uptake and transcytosis of MT, Transferrin (Tf) and PC_3_ as model proteins and peptides, respectively, in the human Caco-2 cell line. We generated specific antibodies against r24p3-R and investigated its expression and localization in rodent and human intestine segments as well as its function in transiently transfected Chinese hamster ovary (CHO) and human Caco-2 BBE cells. We localize 24p3-R to the apical membranes of rodent intestinal mucosa, where it increases in abundance aborally. The human homolog hNGAL-R is expressed in human colonic epithelia as well as in human colon-like, but not duodenum-like Caco-2 BBE cells. Furthermore, hNGAL-R is responsible for high-affinity binding and RME of the peptide PC_3_ as well as apical-to-basolateral transcytosis of PC_3_, MT and Tf in colon-like Caco-2 BBE cells.

## Experimental Procedures

### Ethics statements

Specimens of human tissue were obtained post-mortem from the pathology archives at Aarhus University Hospital (http://www.en.auh.dk/departments/cancer+and+inflammation+centre/department+of+histopathology/research). Studies on this tissue were performed after approval from the local ethical committee, Aarhus County, Denmark.

All animal work has been conducted according to relevant national and international guidelines. Animal housing conditions and experiments were approved by the Danish Ministry of Justice. Experiments on mice were carried out in strict accordance with state health and ethical regulations. Care of animals was in accordance with institutional guidelines. The protocol for sacrifice of animals was approved by the Committee on the Ethics of Animal Experiments of the University of Freiburg (permit number X-07/27A).

## Materials

MT (rabbit apo-MT-2) was from IKZUS Proteomics ( Ge nova , Italy). MT was labelled with Alexa Fluor 546 (A546) as previously described [24]. Alexa Fluor 488 (A488)-PC_3_ and apo-PC_3_ were from Anaspec (Fremont, CA, USA). Human lipocalin-2/(Neutrophil Gelatinase-Associated Lipocalin (NGAL)) was obtained from Enzo Life Sciences (Lörrach, Germany). Lipofectamine 2000 was from Invitrogen (Darmstadt, Germany). 3-(4,5-dimethylthiazol-2-yl)-2,5-diphenyl-tetrazolium bromide (MTT) and protease inhibitor cocktail (PIC) were purchased from Sigma Aldrich (St. Louis, MO; USA). A546-carboxylic acid succinimidyl ester and A546-Tf from human serum were from Molecular Probes Europe BV (Invitrogen, Darmstadt, Germany). MT was coupled to A546, as previously reported [29].

### Antibodies

Polyclonal antibodies were generated in rabbits against the rat peptide sequence for rodent lipocalin-2/r24p3 receptor (r24p3-R) (GenBankTM accession number NP_803156.2) and affinity purified. The epitope sequences for r24p3-R COOH-terminal (α-CT-24p3-R) and NH2-terminal (α-NT-24p3-R) antibodies were CDHVPLLATPNPAL and GALPPNASGWEQPPNSC, respectively. The antibody α-NT-24p3-R detects an extracellular epitope of r24p3-R. The human full-length cDNA of hNGAL-R contains a single open reading frame of 538 amino acids (accession no. Q8WUG5) and pBLAST sequence alignments revealed 100% identity with the α-NT-24p3-R and α-CT-24p3-R peptide sequences. Hence, both antibodies can be used for immunoblotting (1:500) and immunostaining of human and rodent tissues and have been previously characterized [24]. A rabbit polyclonal antibody against the α1-subunit of Na^+^,K^+^-ATPase (Cell Signaling Technology; 1:500), a mouse polyclonal anti β-actin antibody (1: 5,000 v/v; Sigma-Aldrich) and a rabbit polyclonal anti DMT-1 antibody (1: 500 v/v [30]) were used for immunoblotting. Horseradish peroxidase (HRP) -coupled anti-rabbit or anti-mouse IgG (GE Healthcare, Europe GmbH, Munich, Germany) were used as secondary antibodies for immunoblotting and diluted 1:1000-2000. A488-conjugated anti-rabbit IgG (Molecular Probes) and indocarbocyanine (Cy3)-coupled anti-rabbit IgG (Jackson ImmunoResearch Laboratories) were used for immunofluorescence staining and confocal microscopy.

### Immunofluorescence labeling of mouse and human tissue sections combined with confocal laser scanning microscopy

Human tissues were immersion-fixed with 4% formaldehyde in isotonic PBS, pH 7.4. Female SV129 mice (Taconic Europe) were housed under standard conditions and received a standard rodent chow and free access to water. Mice were perfused with 3% paraformaldehyde in PBS (PFA-PBS) via the heart, segments of the gastrointestinal tract were dissected and thoroughly flushed with several changes of ice-cold PBS and post-fixed for 2h at 4^o^C in 0.3% PFA-PBS before being processed for embedding in paraffin. Tissue preparation, sectioning and labeling was performed as previously described [31]. The primary antibody (α-CT-24p3-R) was used at a final concentration of 0.2µg/ml. Goat anti-rabbit Alexa488 conjugated secondary antibodies (Invitrogen) and a TOPRO3 nuclear stain were used for visualization of labeling. Control experiments were performed using omission of primary antibody, secondary antibody or pre-incubation of primary antibody with a 10-fold molar excess of synthetic peptide followed by incubation overnight at 4^o^C, before application to tissue sections. Sections were mounted using glycerol-based mounting medium containing anti-fade reagent (DAKO). A Leica TCS SL confocal microscope with an HCX PL APO 63× oil objective lens (numerical aperture: 1.40) was used for imaging of labelled sections.

### Immunofluorescence microscopy of Mouse Intestine

C57/BL6 adult mice were anesthetized with ketamine (90mg/kg body weight) and xylazine (12mg/kg body weight) and transcardially perfused with 4% PFA/PBS. Small intestine (duodenum, jejunum, ileum) and the descending colon were excised, and postfixed in 4% PFA overnight at 4° C. Tissue blocks were cryoprotected and frozen in liquid N_2_. Immunofluorescence was performed on 5µm cryosections [32]. Sections were rehydrated in PBS, treated with 1% sodium dodecyl sulfate (SDS) for 5 min, blocked with 1% bovine serum albumin (BSA)/ PBS for 15min, and incubated with primary antibody (α-CT-24p3-R) overnight at 4° C at dilution 1:1000. Slides were washed, incubated with donkey anti-rabbit IgG coupled to CY3 (1:600) for 1h at RT, washed, mounted with Vectashield, and viewed with a Zeiss Axioplan2 fluorescence microscope with ApoTome module (Jena, Germany).

### Immunohistochemistry

Following animal euthanasia, segments of mouse or rat gastrointestinal tract were dissected and thoroughly flushed with several changes of ice-cold PBS. Subsequently, tissue was immersion fixed in 4% paraformaldehyde in PBS (PFA-PBS) for 2h at room temperature, followed by 4^o^C overnight in 0.3% PFA-PBS. Tissue was subsequently processed for embedding in paraffin. All procedures for tissue processing and immunohistochemistry have been described in detail previously [33]. The primary antibody (α-CT-24p3-R) was used at a final concentration of 0.4µg/ml. Pre-incubation experiments were performed by combining primary antibody with a 10-fold molar excess of synthetic peptide followed by incubation overnight at 4^o^C, before application to tissue sections. Labeling was visualized by use of a peroxidase-conjugated secondary antibody for light microscopy (P448, Dako, Glostrup, Denmark) and visualized with 0.05% 3,3′-diaminobenzidine tetrachloride (Kemen Tek, Copenhagen, Denmark). Light microscopy was carried out with a Leica DMRE (Leica Microsystems).

### Isolation of rodent gastrointestinal mucosa membranes

Male Sprague-Dawley rats (200-225 g) or female C57BL/6N mice (15-17g) were fasted for 18 h and humanely killed by CO_2_ anesthesia according to the ethical guidelines of the German state on animal welfare (approval no. 50.8735.1 Nr99/4). Segments of duodenum, jejunum, ileum and distal colon were immediately removed and stored on ice in Petri dishes filled with KRH (120 mM NaCl, 4.7 mM KCl, 1.2 mM MgCl_2_, 1.2 mM KH_2_PO_4_, 10 mM HEPES, 10 mM glucose with pH 7.4), supplemented with 0.2 mM Pefabloc SC (Biomol, Hamburg, Germany). Intestinal segments were cleaned, slit open to expose the mucosa and the luminal surface was scraped with a glass cover-slip. The mucosa was transferred into ice-cold 500 µl PBS supplemented with 1:50 PIC, washed 3 times with 500 µl PBS/PIC and resuspended in 250 mM sucrose buffer (250 mM sucrose, 40 mM MOPS, 0.1 mM EGTA, 0.1 mM MgSO_4_ with pH 7.4) with 1:50 PIC prior to homogenization by sonication on ice. To obtain a membrane fraction, homogenate was centrifuged at 233,000 g for 30 min at 4° C and the membrane pellet was resuspended in sucrose buffer/PIC. The protein concentration was determined by Bradford assay [34].

### Cell Culture

CHO-K1 (Chinese hamster ovary; < passage 50) cells (provided by Dr. H. Koepsell, Institute of Anatomy and Cell Biology, University of Würzburg, Germany) were transiently transfected with pcDNA3.1 or rat r24p3-R and cultured as previously described [24]. Caco-2 BBE cells (Human epithelial colorectal adenocarcinoma cells between passage 30-50) were provided by Dr. Didier Merlin, Department of Medicine, Emory University, Atlanta, USA, and cultured in DMEM/F12 medium supplemented with 10% fetal bovine serum (FBS), 60 µg/ml penicillin/streptomycin, 150 ng/ml plasmocin and 1.2 mg/ml NaHCO_3_ and passaged every week before reaching more then 80% confluence. Unless otherwise indicated cells were grown 48 h prior to experiments. To differentiate Caco-2 BBE colon-like cells into a duodenum-like form, cells were grown for 1 week to total confluence and kept at this stage for additional 2 weeks with a medium exchange every 3 days [35–37]. Differentiation into the duodenal-like form was determined by RT-PCR for human DMT-1 (Figure S1).

### Isolation of Caco-2 BBE plasma membranes

Plasma membranes from Caco-2 BBE cells were obtained by differential ultracentrifugation at 4° C, as described previously [24]. The protein concentration was determined by Bradford assay [34].

### Reverse Transcription-Polymerase Chain Reaction (RT-PCR)

cDNA was synthesized from 2 µg of total cellular RNA, and RT-PCR was carried out with MaximaTM Hot Start Green PCR master mix (Fermentas). Primers, GenBank accession numbers, cycling protocols and product sizes are shown in Table S1. Primers for human GAPDH were selected according to Cui et al. [38].

### Immunoblotting

For analysis of homogenate and plasma membranes from Caco-2 BBE cells 7.5 µg of protein was loaded onto the gel. For detection of proteins from rodent mucosal homogenate or membranes 50 µg of total protein was used for detection of hNGAL-R, 10 µg for β-actin and 10 µg for DMT-1 as a duodenal marker (data not shown). Proteins were subjected to 7.5% SDS-PAGE and immunoblotted as described previously [24].

### Live Immunofluorescence Staining of Caco-2 BBE Cells

Experiments with Caco-2 BBE cells were performed with non-permeabilized cells to stain only for hNGAL-R expressed in the cellular plasma membrane, as described previously [24]. Briefly, colon- or duodenum-like Caco-2 BBE cells were seeded at a density of 50,000 cells/coverslip and grown for 48 h prior to staining with primary α-NT-24p3-R antibody (1:100) and secondary Cy3-coupled anti-rabbit IgG (1:600).

### Immunofluorescence Microscopy of Cultured Cells and Quantification of Internalization of Fluorescence-labelled Ligands

The full-length clone of rat r24p3-R was transiently transfected into CHO cells 24 h after seeding using Lipofectamine 2000, as described previously [24]. Forty eight hours after transfection, r24p3-R- and empty vector (pcDNA3.1)-transfected cells were incubated for 24 h with 0-2.8 µM A488-PC_3_, as described previously for A546-MT [24]. Colon- or duodenum-like Caco-2 BBE cells were seeded at a density of 50,000 cells/coverslip and grown for 48 h before addition of different concentrations of A488-PC_3_ or A546-MT ± hNGAL for 24 h in FBS-free medium. Cells were stained with primary α-NT-24p3-R antibody (1:100) and secondary Cy3-coupled anti-rabbit IgG (1:600) for A488-PC_3_ uptake or with A546-conjugated anti-rabbit IgG (1:600) for A546-MT uptake.

For each experiment, 5-10 images were acquired and quantified as described earlier [39]. For determination of r24p3-R-dependent fluorescence uptake in CHO cells, values in vector-transfected cells were subtracted from values obtained in the corresponding r24p3-R-transfected cells. In colon-like Caco-2 BBE cells, heterogeneity of expression was observed with clusters of Caco-2 BBE colon-like cells expressing hNGAL-R whereas other areas did not express hNGAL-R on their surface or showed low levels of expression (Figure S2). Hence, uptake of A488-PC_3_ and A546-MT was analyzed separately in Caco-2 BBE cell domains with high or low levels of hNGAL-R to distinguish between hNGAL-R-dependent and -independent internalization. To determine hNGAL-R-specific uptake of fluorescent dye-labeled proteins in Caco-2 BBE cells 5 images of fields with clusters of hNGAL-R expressing Caco-2 BBE cells and 5 images of clusters of Caco-2 BBE cells not expressing hNGAL-R were analyzed in the absence or presence of the ligand of the hNGAL-R, hNGAL/lipocalin-2 (500 pM).

### Quantification of hNGAL-R-mediated Transcytosis of Fluorescence-labelled Ligands

For transyctosis studies Costar Transwell® culture inserts placed on 24-well plates were used (0.4 µM PE membrane, 0.33 cm^2^ filter area. Corning Inc., Corning, NY, USA). Briefly, 1.3 ml of Caco-2 BBE medium with 10% FBS was added to the basolateral compartment and Caco-2 BBE colon-like cells were plated at a density of 5 x 10^4^ cells/insert in 400 µl medium with 10% FBS added to the apical compartment. The transepithelial electrical resistance (TEER) across the cell monolayer was measured after 0, 24, 48, 72 h culture as well as before and after each experiment. The medium was replaced after 48 h on both, top (apical) and bottom (basolateral) compartments of the culture inserts. The cells were grown in total for 3 days prior to experimentation because preliminary experiments (data not shown) revealed that after 3 days no further change in TEER occurred. Overall, filters with resistance values of 219.72 ± 19.02 Ω*cm^2^ (n = 46; mean ± SD) were used. Before each experiment, the apical medium was replaced by 400 µl medium containing 1% FBS medium and the appropriate fluorescence-labelled ligand at a final concentration of 700 nM with or without 500 pM hNGAL whereas the basolateral medium with 10% FBS remained unchanged. At each experimental time point, 10 µl of the basolateral and the apical medium was removed, frozen at -80° C and subsequently analyzed for fluorescence intensity using standard capillaries in a Monolith NT.015 (NanoTemper Technologies GmbH, Munich, Germany). Data were quantified by semi-logarithmic plotting against standards obtained from stock solutions of fluorescent ligands. The apical decrease of the concentration of fluorescence-labelled ligands at individual time points was plotted by subtracting the respective initial concentration at 0 h in the apical compartment from the measured concentrations of fluorescent ligand. The respective apical decrease and basolateral increase of the concentration of fluorescent ligands were integrated over a period of 8 h and plotted as “area under the curve” (AUC) to express the magnitude of the total changes of ligand concentration in the apical and basolateral compartments over 8 h.

### Microscale Thermophoresis

Binding interactions between colon-like Caco-2 BBE plasma membrane vesicles and the ligand A488-PC_3_ were measured using microscale thermophoresis (MST) on a Monolith NT.015 (Nano-Temper Technologies GmbH, Munich, Germany), as described previously [24,40,41] with the following modifications. Briefly, fixed amounts of colon-like Caco-2 BBE plasma membrane vesicles were pre-incubated for 5 min at room temperature in the dark with different A488-PC_3_ concentrations to achieve ligand-receptor binding equilibrium, or co-incubated with 500 pM hNGAL as a binding competitor to A488-PC_3_. Plasma membrane vesicles and ligand were diluted into the recommended MST Optimized Buffer (50mM Tris pH 7.4, 150mM NaCl, 10mM MgCl_2_, 0.05% Tween-20). All measurements were performed in standard capillaries (Nano-Temper Technologies GmbH) at room temperature. A binding curve was obtained by plotting normalized fluorescence values (*F*_*norm*_) at a given time against varying A488-PC_3_ concentrations and *EC*_*50*_ values were obtained by fitting the binding curves with the Hill equation assuming a Hill coefficient of 1.0. No change in the thermophoretic properties was observed when A488-PC_3_ fluorescence ± 500 pM hNGAL was measured in the absence of membrane vesicles, which served as a negative control (data not shown).

### Statistics

Unless otherwise indicated, the experiments were repeated at least three times and means ± S.D. were calculated, unless otherwise indicated. Analysis by unpaired Student’s t test or one-way ANOVA assuming equality of variance with Newman-Keuls Multiple Comparison test and Dunnett’s post-hoc test was performed with GraphPad Prism 5.0. A488-PC3 uptake in r24p3-R transfected CHO cells followed a one-site binding hyperbolic curve fitting function, whereas uptake in colon-like Caco-2 BBE cells displayed a biphasic two-site binding hyperbolic curve progression. Transcytosis data fitted using a one-phase exponential decay function. All curve fits were done with GraphPad Prism 5.0. Results with P < 0.05 were considered to be statistically significant.

## Results

### 24 p3-R/hNGAL-R is expressed apically in mammalian intestine

**Figure 1:**
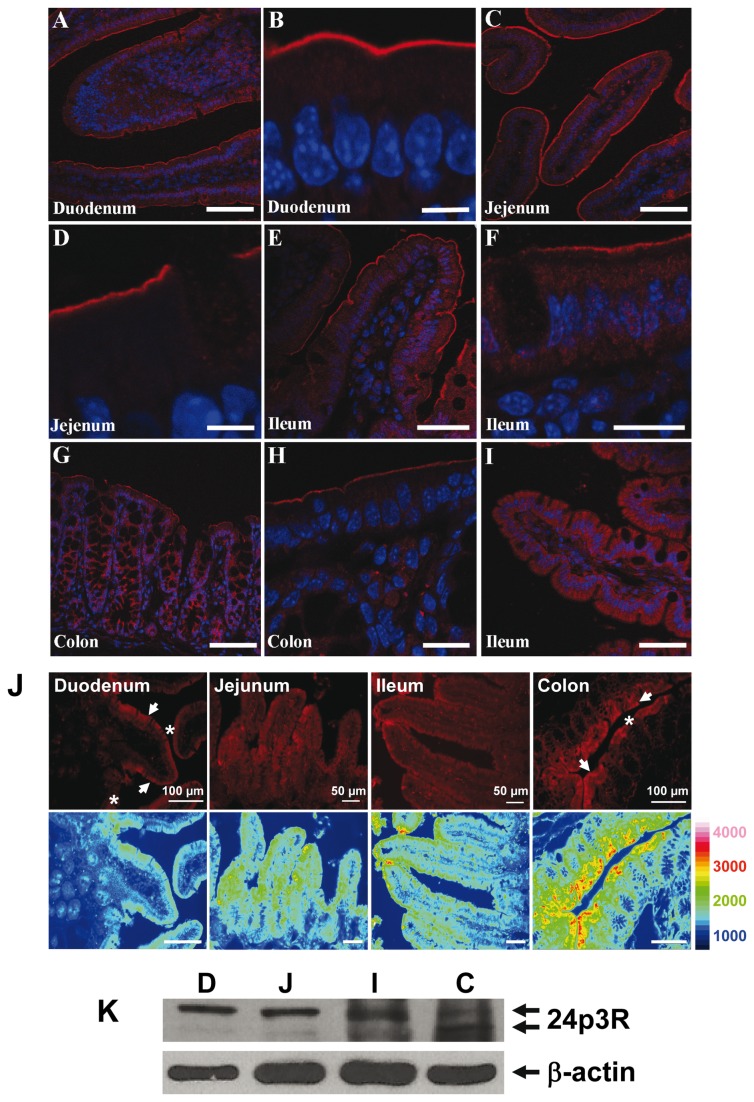
Localization of r24p3-R in mouse intestine. (**A**–**I**) Confocal laser-scanning microscopy of tissue sections labelled with r24p3-R. Red = r24p3-R, blue = nuclei. r24p3-R labeling was associated with the luminal brush border in duodenum (**A** and **B**), jejunum (**C** and **D**), ileum (**E** and **F**) and colon (**G** and **H**). Even using higher laser intensity, no labeling was detectable in any segment following pre-incubation of r24p3-R antibody with the immunizing peptide as demonstrated in ileum (**I**). Scale bars: A, C, E, G, I = 75µm; B and D = 7.5µm; F and H = 20µm. (**J**) Cellular distribution of r24p3-R in mouse small intestine and colon by immunofluorescence microscopy. r24p3-R immunolabeling was observed at the apical cell side of enterocytes (*arrows*) in the epithelium of duodenum, jejunum, ileum and colon, whereas goblet cells (*asterisks*) were devoid of r24p3-R immunoreactivity. r24p3-R labeling intensity revealed an increasing order duodenum<jejunum<ileum < colon. Blue and red pseudocolors represent the lowest and highest values of labeling intensity on a rainbow scale, respectively. (**K**) Immunoblots of homogenates of different mouse intestinal segments (D = duodenum; J = jejunum; I = ileum; C = colon) were performed with α-CT-24p3-R. A specific double band at ~60 kDa (*arrows*) was detected in all segments, but r24p3-R expression was more prominent in ileum and colon, in accordance with immunohistochemical staining of mouse intestine. For loading controls, the same membranes were reprobed with antibodies to β-actin.

**Figure 2:**
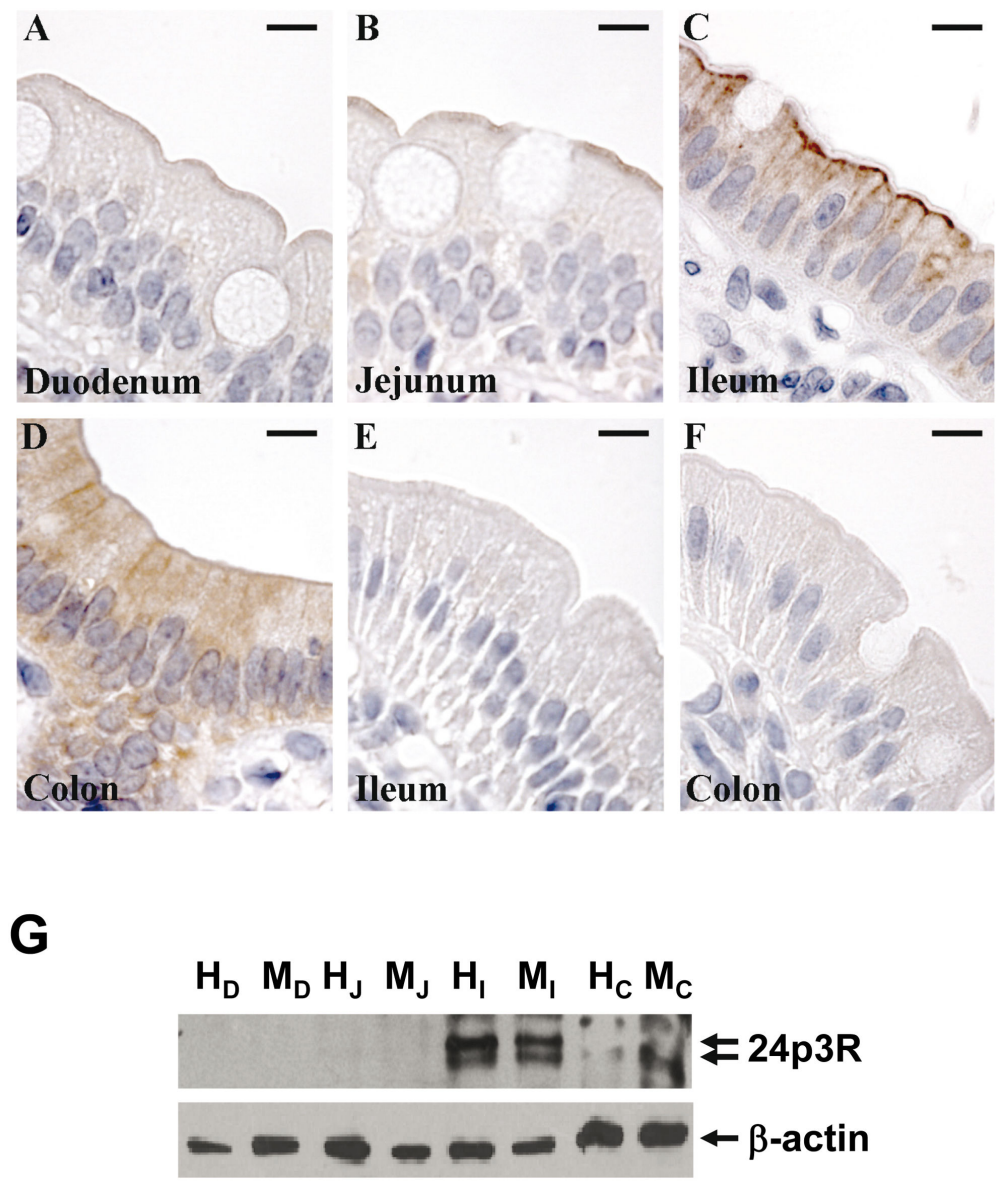
Immunohistochemical labeling of r24p3-R in rat intestine. Very weak r24p3-R immunoreactivity was observed at the apical brush border of duodenum (**A**) and jejunum (**B**). Stronger labeling was observed in the apical brush border of ileum (**C**). Diffuse intracellular labeling was also observed in the ileum and colon (**D**). No labeling was detectable in any segment following pre-incubation of r24p3-R antibody with the immunizing peptide as demonstrated in ileum (**E**) or colon (**F**). Scale bar = 10µm. (**G**) Immunoblots of homogenates (H) and the membrane fraction (M) of different rat intestinal segments (D = duodenum; J = jejunum; I = ileum; C = colon) were performed with α-CT-24p3-R. A specific double band at ~60 kDa (*arrows*) was detected in ileum and colon, in accordance with immunohistochemical staining of rat intestine. For loading controls, the same membranes were reprobed with antibodies to β-actin.

**Figure 3:**
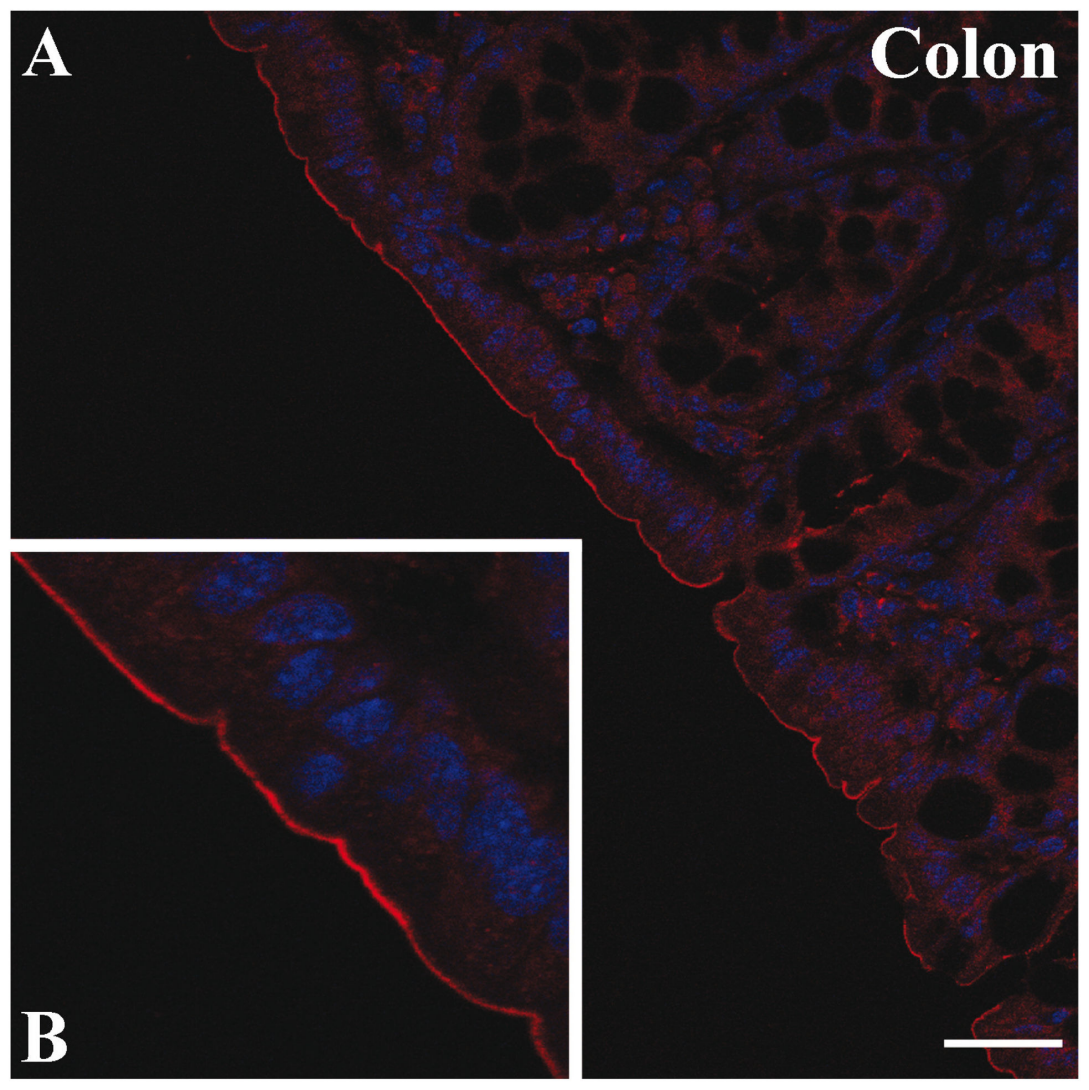
Localization of the human homolog of r24p3-R, hNGAL-R, in human colon. Confocal laser-scanning microscopy of human colon tissue sections labelled with hNGAL-R. Red = hNGAL-R, blue = nuclei. hNGAL-R labeling was predominantly associated with the luminal brush border (**A**). At high magnification (**B**) the apical brush border labeling of epithelial cells is clear. Scale bars: A= 20µm.

Immunolabeling of mouse intestinal segments with a specific antibody against the C-terminal sequence of r24p3-R (α-CT-24p3-R) followed by laser scanning confocal or immunofluorescence microscopy demonstrated specific labeling in all segments of the intestinal tract, including small intestine and colon (Figure 1A–1H; Figure 1J). Labeling was predominantly associated with the surface of enterocytes, which indicates expression of r24p3-R in apical mucosal plasma membranes. No labeling was detected following pre-incubation of r24p3-R antibody with the immunizing peptide, as demonstrated in ileum (Figure 1I). r24p3-R immunoreactivity was restricted to the enterocytes, whereas goblet cells were not labelled. Moreover, r24p3-R labeling intensity increased aborally with highest expression observed in ileum and particularly in colon, as illustrated in Figure 1J, which is similar to mouse kidney, with increasing expression from the proximal to the distal nephron [24]. Indeed, in colon, r24p3-R immunostaining of strong intensity was found in the enterocytes lining the epithelium covering the crypts (*arrows*). Again, mucous cells were devoid of r24p3-R immunoreactivity (Figure 1J, *arrows*). Immunoblots of mucosal homogenate from the different gastrointestinal segments detected a double band at ~60 kDa (Figure 1K), similarly as found in plasma membranes of cells of the distal nephron [24], confirming the greater expression of r24p3-R in ileum and colon (Figure 1K). Similarly, rat intestine showed predominant r24p3-R expression in ileum and colon, as demonstrated by immunohistochemistry (Figure 2A–2F) and immunoblotting of homogenate and membrane fractions (Figure 2G). Expression of the human homolog of r24p3-R, hNGAL-R, was confirmed in human colon (Figure 3). Hence, the apical localization of r24p3-R/hNGAL-R in mammalian intestine suggested its involvement in protein absorption and/or transcytotic processes.

### hNGAL-R is expressed in human colon-like Caco-2 BBE cells

**Figure 4:**
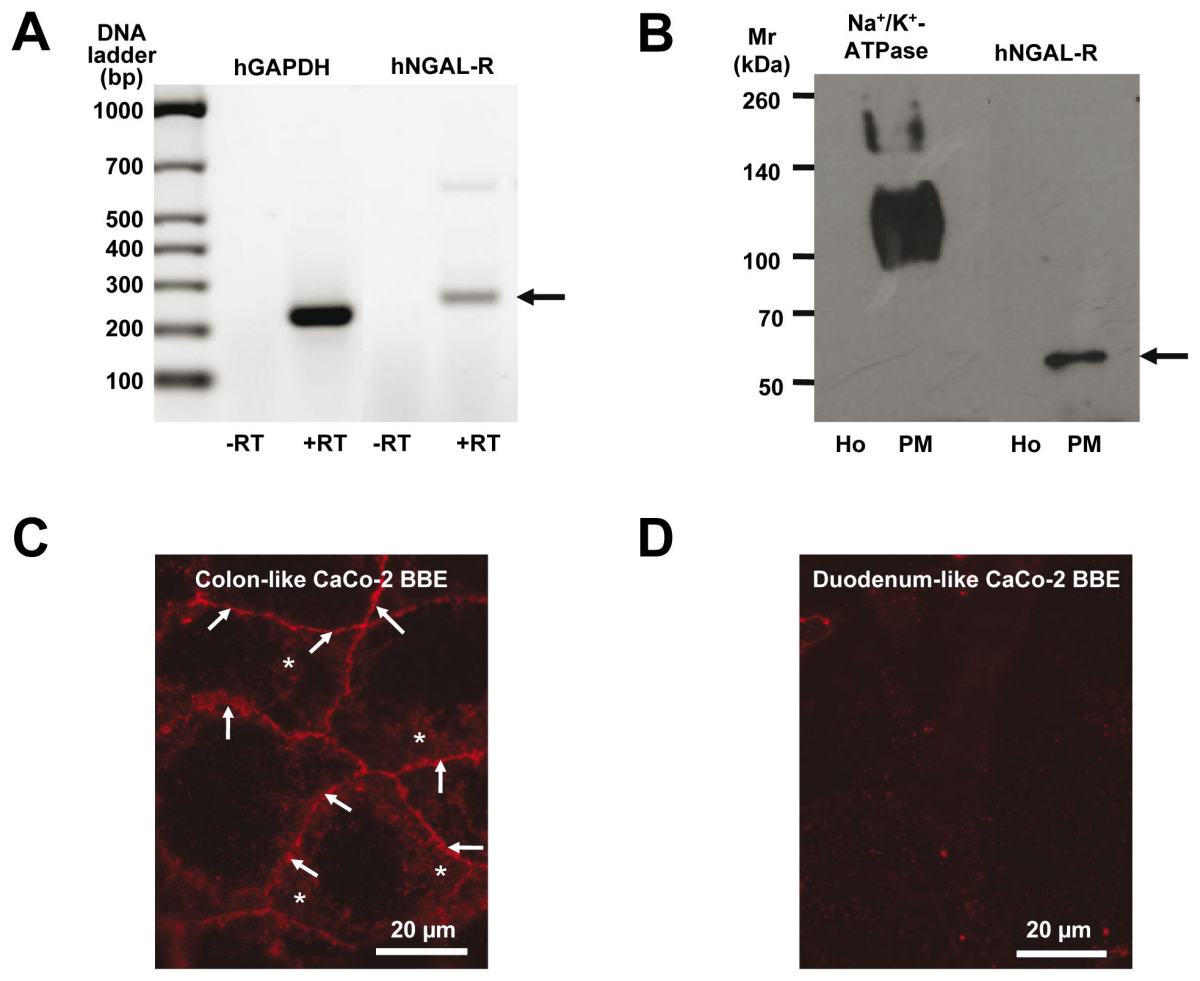
Expression of hNGAL-R in Caco-2 BBE cells. RT-PCR for hNGAL-R and GAPDH in colon-like Caco-2 BBE cells (**A**). A PCR product of 296 bp is amplified from colon-like Caco-2 BBE cell cDNA using specific primers for human NGAL-R and reverse transcriptase (+RT), but not in the control reaction without reverse transcriptase (-RT). The housekeeping gene human GAPDH is used as a control. A 326 bp PCR product is only amplified in the presence of reverse transcriptase (+RT). Immunoblotting of colon-like Caco-2 BBE cell homogenate (Ho) and plasma membranes (PM) (**B**). Specific signals are detected in PM of colon-like Caco-2 BBE cells with antibodies against hNGAL-R (α-CT-24p3-R; 1:500) and the α1-subunit of Na^+^,K^+^-ATPase (1:500). Live immunofluorescence staining of non-permeabilized colon- and duodenum-like Caco-2 BBE cells (**C** and **D**). Immunofluorescence staining with α-NT-24p3-R (1:100) reveals hNGAL-R expression (red fluorescence) at apical (asterisks) and lateral plasma membranes (arrows) of colon-like Caco-2 BBE cells (**C**). No staining for hNGAL-R is detected in duodenum-like Caco-2 BBE cells (**D**).

The human epithelial colorectal adenocarcinoma Caco-2 BBE cell line was selected for further investigation of the function of hNGAL-R. As shown in Figure 4A, the mRNA for hNGAL-R was expressed in human colon-like Caco-2 BBE cells. Plasma membrane localization of hNGAL-R was confirmed by isolation of plasma membranes from human colon-like Caco-2 BBE cells (Figure 4B). Clear signals for hNGAL-R at ~60 kDa and at ~100 kDa for the plasma membrane marker Na^+^,K^+^-ATPase were detected in the plasma membrane fraction, but not in cell homogenate when 7.5 µg protein was loaded onto the gel (Figure 4B). Furthermore, plasma membrane localization of hNGAL-R was confirmed by surface staining of living non-permeabilized colon-like Caco-2 BBE cells with α-NT-24p3-R as shown in Figure 4C (*asterisks*). Strong staining of hNGAL-R at lateral cell sides was also detected (Figure 4C, *arrows*). In contrast, in duodenum-like Caco-2 BBE cells, hNGAL-R was poorly expressed (Figure 4D).

### hNGAL-R mediates internalization of PC_3_ and MT in human colon-like Caco-2 BBE cells

**Figure 5:**
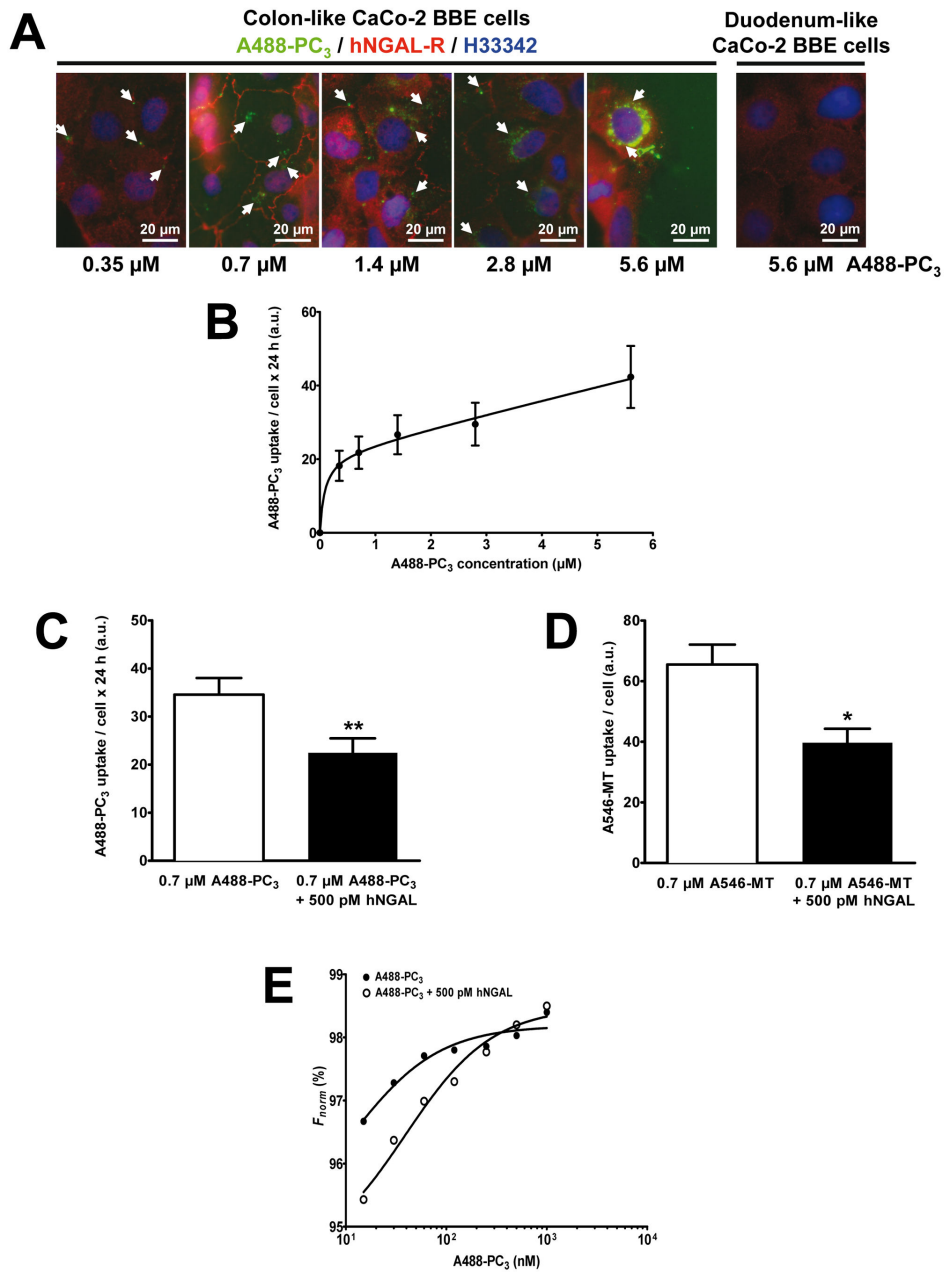
NGAL-R mediates internalization of PC_3_ and MT in colon-like Caco-2 BBE cells. Immunofluorescence microscopy of Caco-2 BBE cells exposed to A488-PC_3_ or A546-MT. Red = hNGAL-R, green = A488-PC_3_, blue = nuclei. Colon-like Caco-2 BBE cells expressing hNGAL-R internalize A488-PC_3_ after 24 h exposure as a function of its concentration (0.35–5.6 µM); in contrast, duodenum-like Caco-2 BBE cells show no expression of hNGAL-R and no uptake of A488-PC_3_ (5.6 µM) after 24 h (**A**). Nuclei are stained with Hoe33432. Concentration-dependence of A488-PC_3_ uptake in colon-like Caco-2 BBE cells is biphasic, suggesting a high- and low-affinity internalization process (**B**). Means ± SD of 5-6 experiments are shown. Uptake of 0.7 µM A488-PC_3_ by colon-like Caco-2 BBE cells is partly mediated by hNGAL-R because it is significantly blocked by 500 pM hNGAL (means ± SD of 5 experiments; ** *P*<0.01) (**C**). Internalization of 0.7 µM A546-MT by colon-like Caco_2_-BBE cells is also partly blocked by 500 pM hNGAL (means ± SD of 4 experiments; * *P*<0.05) (**D**). A488-PC_3_ binding to plasma membrane (PM) vesicles of colon-like Caco_2_-BBE cells using microscale thermophoresis (**E**). The normalized fluorescence (*F*_*norm*_ in %) of A488-PC_3_ (10-1000 nM) bound to colon-like Caco-2 BBE PM vesicles (*full circles*) shows a right shift by co-incubation with 500 pM hNGAL (*open circles*) indicating a reduced binding affinity. The experiment is representative of three similar ones.

To investigate whether PCs of plant origin may be internalized by colonic epithelia by receptor-mediated endocytosis, we incubated human Caco-2 BBE cells for 24 h with increasing concentrations of A488-PC_3_ (up to 5.6 µM) for 24 h (Figure 5A). A488-PC_3_ was internalized as a function of its concentration and data were best fit assuming two-site binding to Caco-2 BBE cells with a first _*app*_ *K*_*D*_ at ~90 nM and a second _*app*_ *K*_*D*_ at ~300 nM (Figure 5B). In contrast, duodenum-like Caco-2 BBE cells showed weak uptake following exposure to 5.6 µM A488-PC_3_ for 24 h. The specificity of A488-PC_3_ uptake in colon-like Caco-2 BBE cells was further demonstrated by co-incubation of A488-PC_3_ with the high-affinity ligand of hNGAL-R, hNGAL, at a saturating concentration of 500 pM ( _*app*_ *K*_*D*_ ~92 pM) to block A488-PC_3_ internalization [42]. Uptake of 0.7 µM A488-PC_3_ by colon-like Caco-2 BBE cells after 24 h incubation was significantly reduced by addition of 500 pM hNGAL (Figure 5C). These data indicated that colon-like Caco-2 BBE cells internalize PC_3_ partly via hNGAL-R, but another endocytosis mechanism that is not blocked by 500 pM hNGAL also contributes to PC_3_ internalization in Caco-2 BBE cells (possibly the multi-ligand receptor megalin, which has an almost 1000-fold lower affinity to hNGAL [43] and is also expressed in Caco-2 BBE cells; data not shown). CHO cells transiently transfected with r24p3-R, the rat homolog of hNGAL-R, but not vector-transfected CHO cells, showed concentration-dependent uptake of A488-PC_3_, similarly as previously shown for MT, Tf and albumin [24], confirming lipocalin-2/r24p3/hNGAL receptor-specific endocytosis of A488-PC_3_ (Figure S3). Similarly, uptake of 0.7 µM A546-MT by colon-like Caco-2 BBE cells after 24 h incubation was partly reduced by addition of 500 pM hNGAL (Figure 5D). Overall, these data indicate that colon-like Caco-2 BBE cells internalize PC_3_ and MT partly via hNGAL-R. Finally, steady-state binding of A488-PC_3_ to Caco-2 BBE cell plasma membranes vesicles was determined by MST [24,40,41] and essentially confirmed the data obtained in intact cells (Figure 5E). An *EC*_*50*_ of 18.6 ± 12.2 nM (Mean ± SD of 3-4 experiments) for binding of A488-PC_3_ to membrane vesicles was determined. Furthermore, co-incubation with 500 pM hNGAL reduced binding affinity (EC_50_ = 224.9 ± 353.4 nM; mean ± SD of 3-4 experiments) (Figure 5E).

### hNGAL-R-mediated transcytosis of PC_3_, MT and Tf in colon-like Caco-2 BBE cells

**Figure 6:**
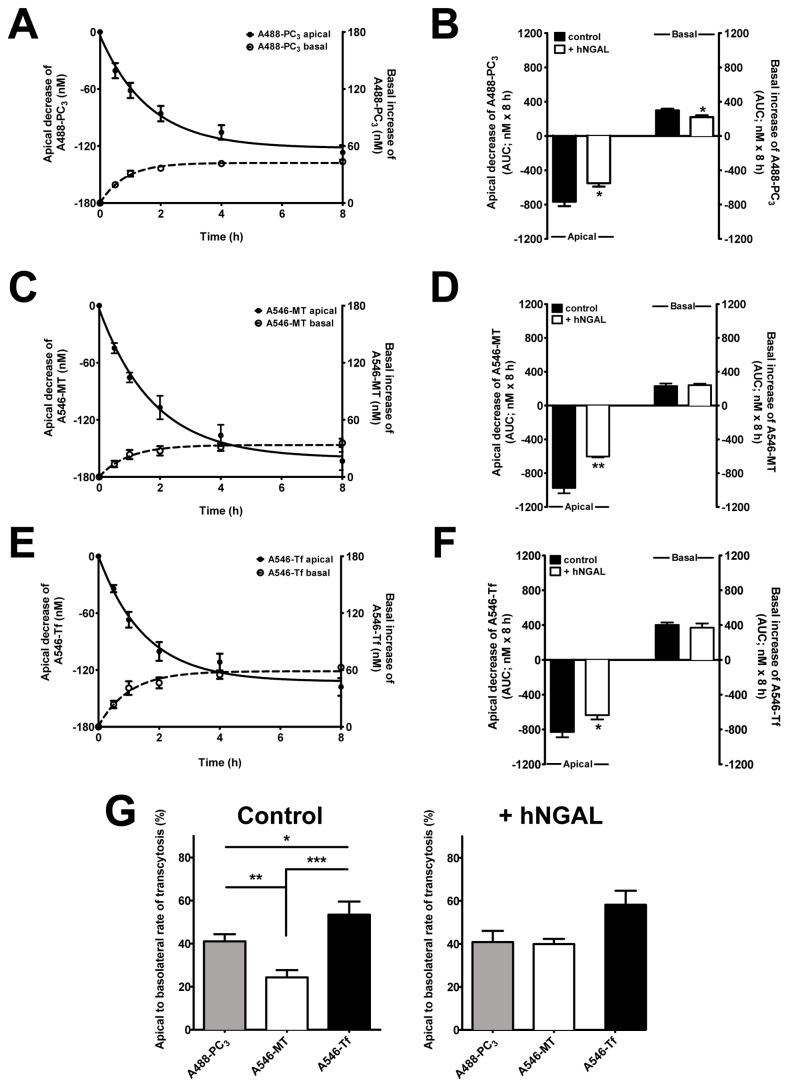
NGAL-R mediates apical-to-basolateral transcytosis of PC3, MT and Tf in confluent Caco-2 BBE cell monolayers. Transcytosis of fluorescence-labelled ligands through confluent monolayers of Caco-2 BBE cells plated on Transwell culture inserts. Fluorescent ligands of hNGAL-R were added to the apical compartment at a final concentration of 700 nM and the apical decrease as well as the basolateral increase of the concentration of the fluorescent ligands was recorded over a period of 8 h (**A**, **C** and **E**). Confluent colon-like Caco-2 BBE monolayers display rapid NGAL-R dependent apical-to-basolateral transcytosis of A488-PC3 (**A**), A546-MT (**C**) and A546-Tf (**E**), which saturates at 4-8 h. Means ± SEM of 9-14 experiments are shown. Data are fitted to a one-phase exponential decay function. For further details, see Experimental Procedures. Experiments with or without 500 pM hNGAL in the apical compartment were also performed to determine the contribution of hNGAL-R to transcytosis (**B**, **D**, **F** and **G**). To obtain a quantitative estimate of the apical decrease and basolateral delivery of the fluorescent ligands due to transcytosis and to determine the impact of hNGAL on this process experimental data were integrated over a period of 8 h and expressed as “area under the curve” (AUC) (**B**, **D** and **F**). hNGAL significantly reduces the apical decrease of fluorescent ligand concentration. Means ± SEM of 9-14 experiments are shown; * *P*<0.05; ** *P*<0.01. A plot of the ratio of basolateral to apical AUC of fluorescent PC_3_, MT and Tf, as shown in B, D and **F**, provides an estimate of transcytosis efficiency as well as the proportion of intracellular ligand trapping in the absence or presence of 500 pM hNGAL (**G**). In controls, intracellular A546-MT trapping is significantly increased compared to A488-PC_3_ or A546-Tf, but not with hNGAL. The data are expressed as a percentage of apical AUC. Means ± SEM of 9-14 experiments are shown; * *P*<0.05; ** *P*<0.01; *** *P*<0.001.

Previous reports have indicated that MT and PC [8,9] and other proteins, such as HIV or immunoglobulins [3,18,44], may be taken up by the intestine in intact form. Moreover, transcytosis of Tf has been demonstrated in Caco-2 cells [11,17,45]. We have previously demonstrated that MT and Tf are ligands of r24p3-R/hNGAL-R [24], therefore, we investigated whether hNGAL-R-mediated uptake of PC_3_, MT and Tf is associated with transcytosis of these proteins in colon-like Caco-2 BBE cells. Transwell filters with colon-like Caco-2 BBE monolayers with a TEER of 200-250 Ω*cm^2^ were incubated with 700 nM A488-PC_3_, A546-MT or A546-Tf in the apical compartment for 8 h. The TEER remained unchanged throughout the experimental period (data not shown). As shown in Figure 6A, 6C and 6E, the ligand concentration in the apical compartment rapidly decreased after 0.5 h but remained constant after 4-8 h, which was reflected by a similar kinetics of increase of ligand concentration in the basal compartment (this apical-to-basolateral delivery of fluorescent ligands was inhibited by the cytoskeleton disrupting agent nocodazole, indicating that ligands were indeed crossing the epithelium via transcytosis; manuscript in preparation). Notably, the magnitude of the decrease of A488-PC_3_ and A546-Tf concentrations in the apical compartment was comparable whereas more A546-MT was removed from the apical compartment (compare Figure 6A and 6E with Figure 6C). Co-application of 500 pM hNGAL to the apical compartment significantly reduced overall dynamics of the fluorescent ligand transcytosis (Figure 6B, 6D and 6F), as determined by integration over a period of 8 h and expressed as “area under the curve” (AUC). This data suggests that ligand removal from the apical compartment by colon-like Caco-2 BBE monolayers is partly mediated by the hNGAL-R. Strikingly, inhibition of apical applied fluorescent proteins by hNGAL was more prominent with A546-MT (~40% compared to ~25% for A488-PC_3_ and A546-Tf), suggesting preferential uptake of A546-MT via an hNGAL-R dependent endocytosis pathway in colon-like Caco-2 BBE monolayers, whereas A488-PC_3_ or A546-Tf may be largely internalized via alternative endocytosis pathways (compare Figure 6D with Figure 6B and 6F). In contrast, basolateral delivery of A546-MT was reduced compared to A488-PC_3_ or A546-Tf (e.g. under control conditions 231.2 ± 20.2 nM x 8 h for A546-MT compared to 299.4 ± 18.7 nM x 8 h for A488-PC_3_ or 402.4 ± 28.0 nM x 8 h for A546-Tf; means ± SEM; n = 9-14). This was further supported by calculating the ratio of basal delivery to apical decrease of fluorescent PC_3_, MT and Tf as an indicator of transcytosis efficiency, which showed significantly reduced A546-MT transcytosis compared to A488-PC_3_ or A546-Tf in control cells, but not in the presence of hNGAL and suggesting hNGAL-R dependent intracellular trapping of A546-MT (Figure 6G).

## Discussion

In this study, the r24p3-R/hNGAL-R expression and function in rodent and human intestine was investigated. In rat and mouse intestine, expression of r24p3-R increased aborally (Figures 1 and 2) with a predominant localization at the luminal cell side of the enterocytes in the ileal and colonic mucosa. This was further supported by the surface expression of hNGAL-R in human colon (Figure 3) and in colonic-like Caco-2 BBE cells whereas duodenum-like Caco-2 BBE cells were devoid of hNGAL-R (Figure 4). The preferential distribution of r24p3-R in the distal segments of the intestine is similar to that found in the rodent nephron [24]. The ligand of r24p3-R, Lipocalin-2/r24p3/hNGAL has been shown to play a role as an iron-sequestering protein in the antibacterial innate immune response [26]. It has also been reported that lipocalin-2-deficient mice have increased bacterial infections in the urinary tract [27]. In our recent publication [24], we hypothesized, that the predominant localization of r24p3-R in the inner medullary collecting duct might also be a defence line against ascending infections. In the mammalian /human intestine, the distal segments (ileum, colon and even jejunum) are permanently colonized by bacteria after birth [5,46]. Hence, it can be speculated that Lipocalin-2/r24p3/hNGAL and its receptor may be necessary to control the interaction of the host with indigenous intestinal micro-organisms and/or to protect the host upper intestine against ascending bacterial colonization and infections of the gastrointestinal tract.

Recently, we demonstrated that the lipocalin-2/r24p3/hNGAL receptor mediates high-affinity binding and RME of metal-binding protein as ligands, such as MT and Tf, besides lipocalin-2/hNGAL/r24p3 [24,25,42]. So far, *in vivo* studies have provided limited evidence for expression of the megalin/cubilin/amnionless receptor complex in the terminal ileum [13,14] (reviewed in 15, which plays a role in apical endocytosis of he intrinsic factor-vitamin B12 complex and possibly Tf [16]. Moreover, the Tf receptor may mediate apical uptake and transcytosis of Tf though, in contrast to *in vitro* studies [11,17,18], evidence for apical expression of the Tf receptor *in vivo* is sparse [18]. Here we focussed on the possibility that endo-/transcytosis of proteins, such as Tf, but also MT and PC_3_, may also be mediated by the r24p3-R/hNGAL-R in the intestine. Previous studies had suggested that a significant proportion of MT and PC_3_ reach the intestinal lumen in intact form [9,47,48]. Like other proteins, MT from animal sources is largely digested in the upper intestinal tract, whereas MT and PC from plant origin become available after bacterial fermentation in the lower intestinal tract [5,6]. Cadmium-bound MT and PC are absorbed intact by intestinal enterocytes *in vivo* and can be subsequently found in the host kidney [8,9]. Combining our localization (Figures 1-4) and functional data (Figures 5 and 6; Figure S3), we propose that r24p3-R/hNGAL-R is also responsible for a significant proportion of RME and transcytosis of MT and PC_3_ (and possibly other) proteins in the lower intestine *in vivo*. The affinity of these ligands to r24p3-R/hNGAL-R appears to be high with ~19 nM for A488-PC_3_ binding in colon-like Caco-2 BBE cells (Figure 5E) and ~120 nM for A546-MT binding in renal cells [24]. Together with data from our previous study [24], it suggests that r24p3-R/hNGAL-R is a high-affinity and multi-ligand receptor for protein endocytosis and transcytosis in absorptive epithelia, including kidney and intestine.

The partial inhibition of ligand internalization and transcytosis by hNGAL (Figures 5 and 6) and the presence of a second low-affinity binding site for PC_3_ in colon-like Caco-2 BBE cells (Figure 5B and 5E) suggest that in addition to r24p3-R/hNGAL-R, another receptor/uptake mechanism for PC_3_ is present in colon-like Caco-2 BBE cells. Megalin, which is expressed in colon-like Caco-2 BBE cells (data not shown and [49]), may be responsible for this second binding site.

Transcytosis of the three ligands investigated differed in colon-like Caco-2 BBE cells (Figure 6). Overall, A546-Tf transcytosis was more efficient than that of A488-PC_3_ or A546-MT (Figure 6G). This specific difference could be due to the presence of apical Tf-R in Caco-2 cells [11,17] that may additionally contribute to efficient RME and transcytosis. Furthermore, the magnitude of hNGAL-R-dependent A546-MT uptake was higher than A488-PC_3_ or A546-Tf (Figure 6B, CD and 6F), suggesting that A546-MT is preferentially taken up by the hNGAL-R endocytosis pathway in colon-like Caco-2 BBE cells and/or that other pathways for RME of A546-MT have a lower affinity to this ligand. In contrast, the basolateral delivery of A546-MT was reduced, as reflected by the significantly lower apical-to-basolateral transcytosis efficiency, compared to A488-PC_3_ or A546-Tf (Figure 6G). This indicates that a large proportion of A546-MT (~75%) remains trapped in the cell. In contrast, when hNGAL-R dependent RME of A546-MT was blocked by co-incubation with the high-affinity ligand hNGAL (500 pM), transcytosis efficiency of A546-MT (~40%) did not differ from that of A488-PC_3_ or A546-Tf with or without co-incubation with hNGAL (Figure 6G). This suggests that different receptors for RME are linked to different pathways and modes of intracellular trafficking and transcytosis in colon-like Caco-2 BBE cells. It remains to be investigated which processes underlie the different rates of transcytosis of A546-Tf, A488-PC_3_ and A546-MT via the hNGAL-R.

In summary, this study demonstrates that the lipocalin-2/r24p3/hNGAL-R is expressed in the luminal membranes of mammalian intestinal mucosa, with greater expression in the distal segments; ileum and colon. In a cell culture model of colonic epithelia, the Caco-2 cell line, hNGAL-R mediates high-affinity binding, RME and transcytosis of proteins and peptides, such as Tf, MT and PC_3_. We speculate that the lipocalin-2/r24p3/hNGAL-R may contribute to non-selective apical absorption of intact proteins and peptides by the intestine *in vivo*. We further propose that the predominant expression of lipocalin-2/r24p3/hNGAL-R in distal intestinal segments may control host-microbiota homeostasis.

## Supporting Information

Figure S1 **DMT-1 mRNA expression in colon- and duodenum-like Caco-2 BBE cells.**
In duodenum-like Caco-2 BBE cells a PCR product of 355 bp is amplified as a marker of human DMT1, which is absent in colon-like Caco-2 BBE cells. Without reverse transcriptase (-RT) no PCR product is amplified in both Caco-2 BBE cells lines. As a control for mRNA integrity a 326 bp PCR product for human GAPDH is amplified from colon- and duodenum-like Caco-2 BBE cells.
(TIF)

Figure S2 **Heterogenous expression of hNGAL-R in colon-like Caco-2 BBE cells.**
Red = hNGAL-R, blue = nuclei. Staining of non-permeabilized colon-like Caco-2 cells shows clusters with high hNGAL-R expression whereas other areas show poor or no expression of hNGAL-R at all. Nuclei are stained with Hoe33432.
(TIF)

Figure S3 **r24p3-R mediates uptake of PC_3_ in transiently transfected CHO cells.**
Immunofluorescence microscopy of CHO cells exposed to A488-PC_3_. Red = hNGAL-R, green = A488-PC_3_, blue = nuclei. (A) Internalization of A488-PC_3_ is concentration-dependent in r24p3-R over-expressing CHO cells, but not in pcDNA3.1 transfected CHO cells, which show no r24p3-R expression. (B) In CHO cells over-expressing r24p3-R concentration dependence of A488-PC_3_ internalization is hyperbolic with an _*app*_ *K*_*D*_ of ~500 nM, suggesting one binding site for uptake (means ± SD of 3-4 experiments).
(TIF)

Table S1 **Primer List, including gene bank accession numbers, cycling protocols and PCR product sizes**.
(TIF)
