# Supplementary figures and images for: Expression and Function of the Lipocalin-2 (24p3/NGAL) Receptor in Rodent and Human Intestinal Epithelia
Source: PLoS One. 2013 Aug 5;8(8):e71586. doi: 10.1371/journal.pone.0071586 (PMC3734285; doi:10.1371/journal.pone.0071586)

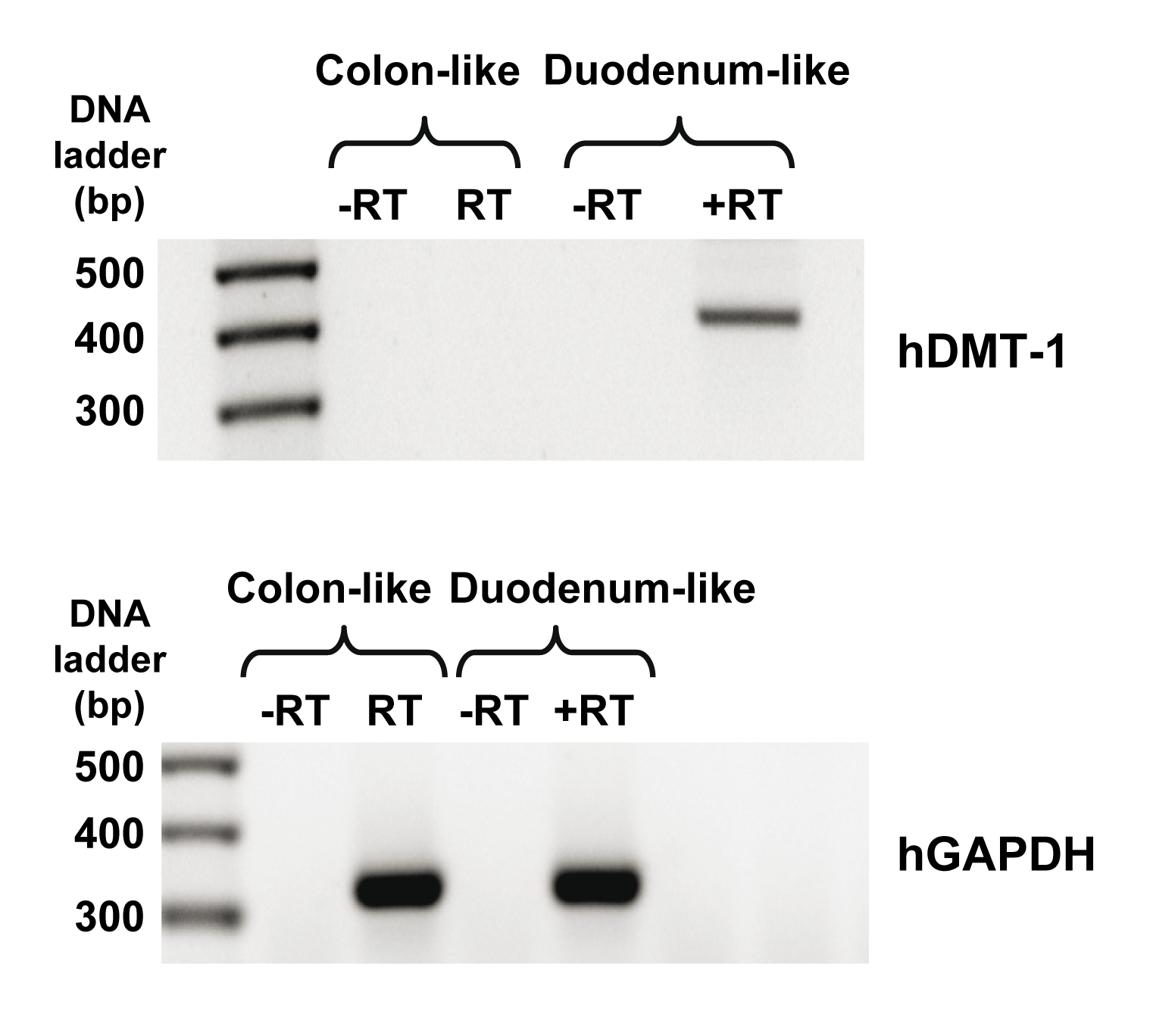

Supplement: Figure S1 — DMT-1 mRNA expression in colon- and duodenum-like Caco-2 BBE cells. In duodenum-like Caco-2 BBE cells a PCR product of 355 bp is amplified as a marker of human DMT1, which is absent in colon-like Caco-2 BBE cells. Without reverse transcriptase (-RT) no PCR product is amplified in both Caco-2 BBE cells lines. As a control for mRNA integrity a 326 bp PCR product for human GAPDH is amplified from colon- and duodenum-like Caco-2 BBE cells. (TIF) [file pone.0071586.s001.tif]

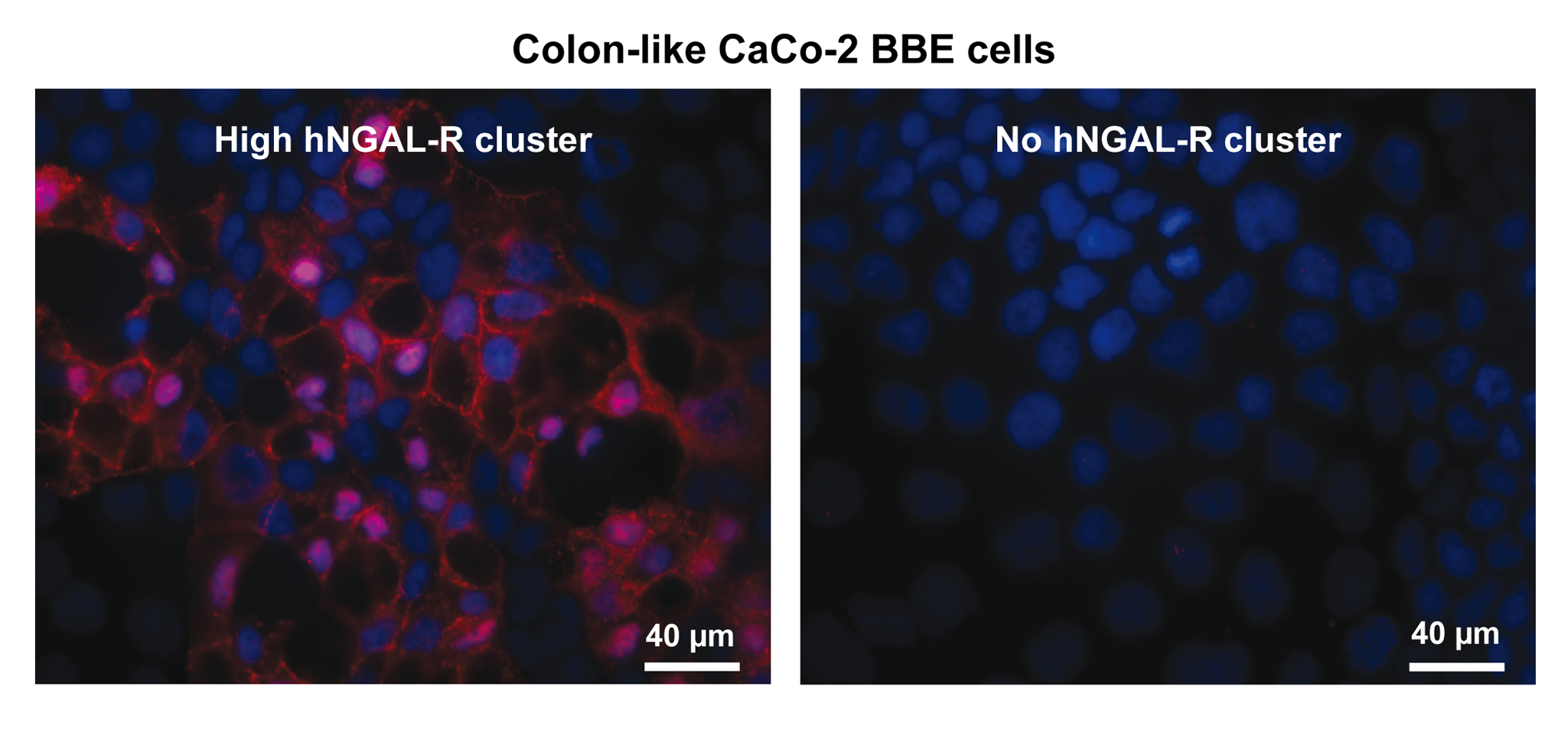

Supplement: Figure S2 — Heterogenous expression of hNGAL-R in colon-like Caco-2 BBE cells. Red = hNGAL-R, blue = nuclei. Staining of non-permeabilized colon-like Caco-2 cells shows clusters with high hNGAL-R expression whereas other areas show poor or no expression of hNGAL-R at all. Nuclei are stained with Hoe33432. (TIF) [file pone.0071586.s002.tif]

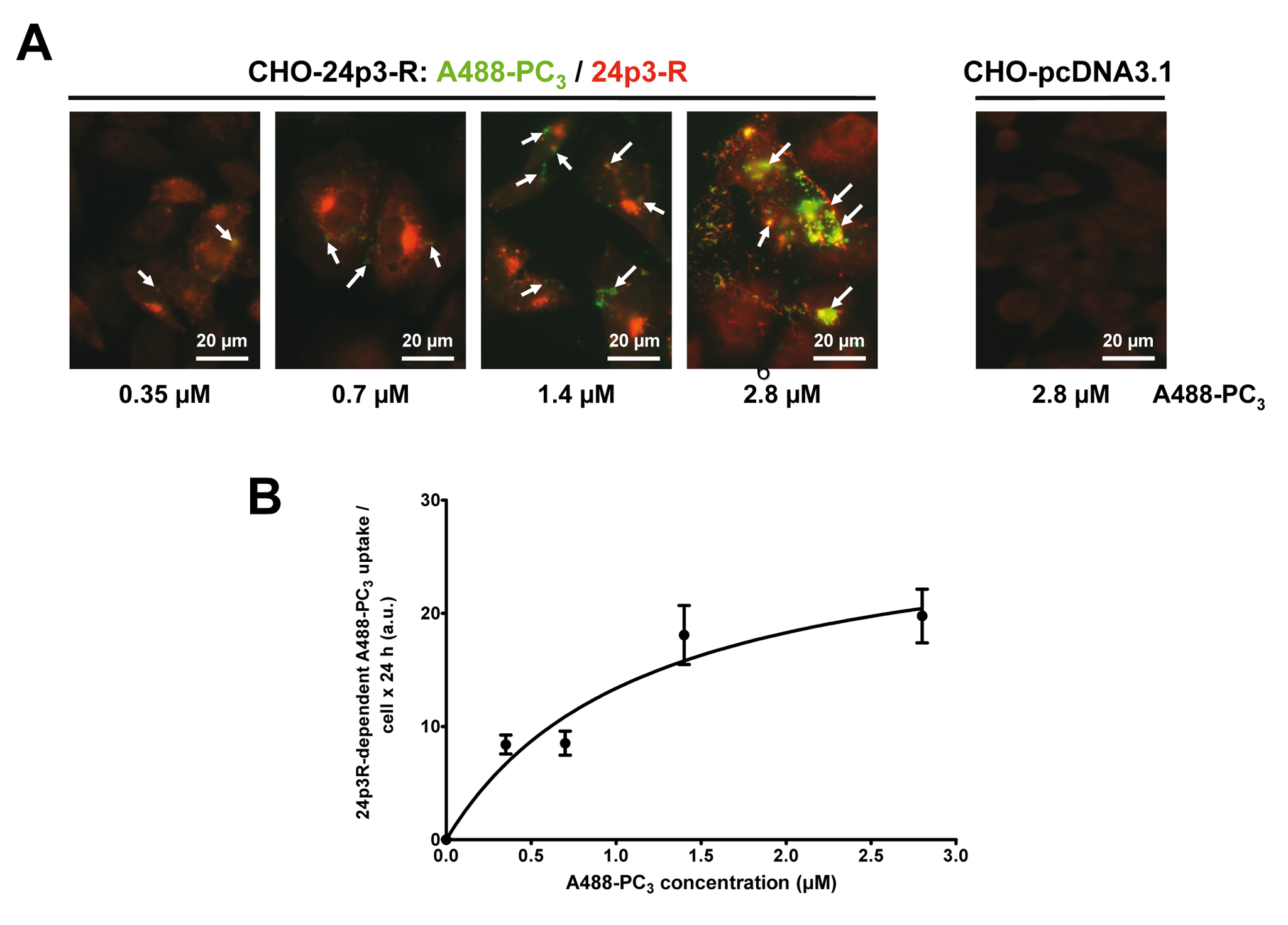

Supplement: Figure S3 — r24p3-R mediates uptake of PC3 in transiently transfected CHO cells. Immunofluorescence microscopy of CHO cells exposed to A488-PC3. Red = hNGAL-R, green = A488-PC3, blue = nuclei. (A) Internalization of A488-PC3 is concentration-dependent in r24p3-R over-expressing CHO cells, but not in pcDNA3.1 transfected CHO cells, which show no r24p3-R expression. (B) In CHO cells over-expressing r24p3-R concentration dependence of A488-PC3 internalization is hyperbolic with an app K D of ~500 nM, suggesting one binding site for uptake (means ± SD of 3-4 experiments). (TIF) [file pone.0071586.s003.tif]

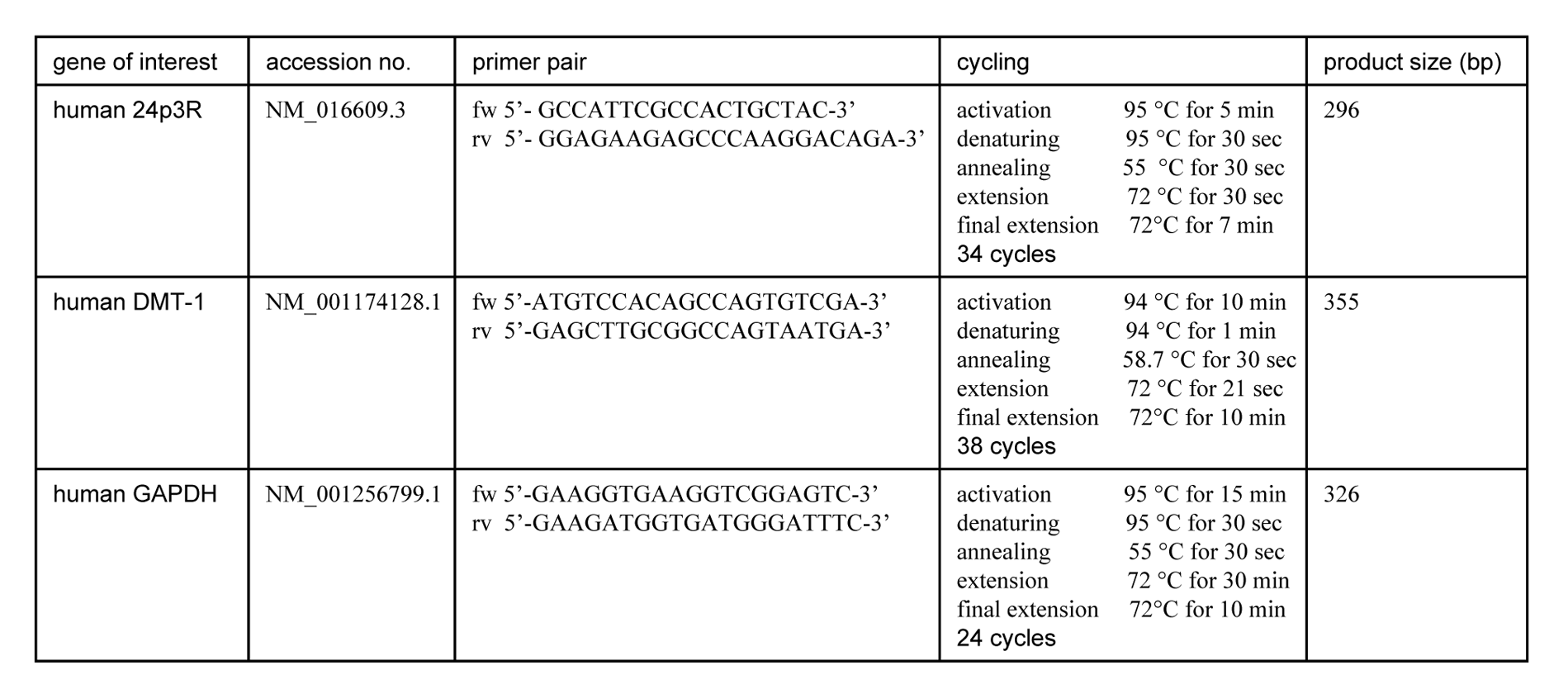

Supplement: Table S1 — Primer List, including gene bank accession numbers, cycling protocols and PCR product sizes. (TIF) [file pone.0071586.s004.tif]
